# COVID-19 in Low and Middle Income Countries

**DOI:** 10.3390/pathogens11111325

**Published:** 2022-11-11

**Authors:** Robert Colebunders, Joseph Nelson Siewe Fodjo

**Affiliations:** 1 Global Health Institute, University of Antwerp, 2610 Antwerp, Belgium; 2 Brain Research Africa Initiative (BRAIN), Yaoundé P.O. Box 25625, Cameroon

The COVID-19 pandemic is by far the worst epidemic in the last century, causing more than 6.56 million deaths so far and affecting all corners of the world [1]. However, this epidemic is very heterogenous and characterized by multiple waves affecting countries and continents with varying rates of mortality. Some low- and middle-income countries (LMICs), including Peru, South Africa, Brazil, and India, were confronted with high COVID-19-related mortality compared to other parts of Latin America, Asia, and Africa [1]. Sub-Saharan Africa (SSA), with its weak health infrastructure, poverty, and the highest rates of neglected tropical diseases [2], was expected to become most affected by the pandemic but rather appears to have been relatively spared compared to the United States of America and Europe [1].

The COVID-19 pandemic also caused a lot of “collateral damage” on the health and education system as well as on the economy, with a very different impact in LMICs [3]. Another side effect of the COVID-19 pandemic is the widespread use of facemasks, which were often poorly disposed of, thus posing an environment threat [4]. 

Very early on in the COVID-19 pandemic, the MDPI journal ***Pathogens*** launched a Special Issue on “COVID-19 in Low- and Middle-Income Countries” to accommodate the massive influx of epidemiological and social science papers. A Special Issue with the same title was also launched in another MDPI journal, the ***International Journal of Environmental Research and Public Health***. In this editorial, we summarize some of the main findings published in these two journals.

To understand the reasons for the heterogeneity of the COVID-19 pandemic, it is important to obtain good quality data about the true level of COVID-19 infection and disease in LMICs. Obtaining such data in most LMICs is problematic because of the low level of COVID-19 testing and case reporting as well as the lack of sero-surveys and a surveillance system for monitoring COVID-19-related hospital admissions and mortality. 

In the Special Issue of *Pathogens*, Feikin et al. proposed a simple method to estimate the percentage of a population infected with SARS-CoV-2 based on COVID-19 death counts in a defined population [5]. This method could provide infection percentages in places where sero-surveys cannot be carried out. However, this method requires access to reliable COVID-19 mortality data. The latter is problematic in most LMICs where COVID-19 deaths are under-counted due to limited testing availability, a lack of clinical suspicion of COVID-19, and suboptimal access to healthcare and testing among pre-fatal cases.

Another simple and low-cost way to obtain rapid information about COVID-19 community transmission at the start of the pandemic was to organize online surveys and ask participants about clinical symptoms suggestive of COVID-19 disease. With the COVID-19 alpha and delta variants, flu-like symptoms together with anosmia strongly suggested a COVID-19 infection. In April 2020, an online survey in Somalia which made use of a clinical definition of COVID-19 infection showed that high COVID-19 community transmission was ongoing in the country, despite only 88 COVID-19 deaths being officially reported at the time [6].

The results of several similar online surveys performed in the first year of the pandemic were presented in the two Special Issues. These surveys also assessed in different LMICs the level of adherence to the COVID-19 preventive measures implemented in these countries. The results revealed important differences between countries.

Adherence to preventive measures in the general public was extremely good in Vietnam [7]. In Vietnam, more than 99% of people reported wearing a face mask when going outdoors [7]. The strict implementation of COVID-19 preventive measures in Vietnam explains the fact that, despite Vietnam being a neighboring country of China, where the pandemic started, no COVID-19 deaths were observed in the first year of the pandemic [1].

Adherence to preventive measures were also found to be relatively good in Morocco [8] and Mozambique [9]. In Morocco, 63.2% of respondents reported that they complied with more than five of nine recommended safety measures, including avoiding going out (93.2%) and frequent handwashing with soap and water (78.2%) [8]. In Mozambique, face-mask use and regular hand washing were reported by more than 90% of the respondents [9]. Additionally, in Cameroon, COVID-19 preventive adherence scores were initially high but gradually decreased over time accompanied by an increasing incidence of symptoms suggesting a COVID-19 infection [10]. This concurs with findings from a population-based study which showed rapidly increasing SARS-CoV-2 seropositivity rates among Cameroonians from 18.6% in January 2021 to 51.3% in April 2021 [11].

In contrast, adherence to COVID-19 preventive measures were sub-optimal in Somalia [12] and Uganda [13]. In Somalia, only half of the respondents reported wearing face masks when going out [12], and in Uganda, this was only 33% [13]. In Cuenca, in Ecuador, despite good overall adherence to the COVID-19 preventive measures by the population, a seroprevalence study showed evidence of high ongoing COVID-19 transmission in certain parishes [14].

In Morocco and Mozambique, a higher adherence with preventive measures was observed with older age, most likely because the COVID-19 mortality risk also increases with age [8,9]. However, this was not observed in the other surveys, perhaps because the majority of participants were young people with mobile phones and an internet connection and also because a high proportion of participants were healthcare workers. 

Adherence to preventive measures was assessed among dental workers and other healthcare workers in Vietnam [15]. Nearly all of them also used face masks outside the healthcare facility [15].

In some of these surveys, the impact of the COVID-19 pandemic on the mental health of the participants was assessed. In Vietnam [15] and Brazil [16], the pandemic had a significant negative impact on the physical and mental health of the healthcare workers. In Vietnam, 37% of dental care workers rated their quality of life during the COVID-19 pandemic as low [15]. In Brazil, 56.6% of healthcare workers screened positive for anxiety, 46.4% for depression, and 36.9% for both conditions [16]. In Western Uganda, 39.8% of pregnant women experienced high psycho-emotional stress related to the pandemic [17].

Online surveys in LMICs have several limitations. Indeed, respondents cannot be considered as a representative sample of the general population since only literate persons with access to the internet were able to participate. Moreover, self-reported responses may not reflect the real-life behavior of participants. Additionally, the online approach makes it impossible to verify the reality of the situation in the field at the time of response. Moreover, in most of the online surveys, a snowball-sampling-based online survey was used. Such a methodology further compounds the selection bias. Indeed, it is possible that the first participants determined the remaining participants, as they shared the link only to their networks. As some surveys were launched in medical schools, this resulted in a large percentage of respondents being medical students and healthcare workers. Such a population is likely to be more aware of and compliant with preventive measures.

AA Hatab et al. carried out a systematic literature review about the COVID-19 impact on livestock systems and food security in developing countries [18]. Their review confirmed that the consequences of the COVID-19 pandemic posed significant stresses to livestock systems in low-income countries and drastically disrupted many activities along livestock supply chains. They concluded that more research is needed on COVID-19’s impact on the livestock supply chains and recommended more holistic, integrated, and resilience-based approaches to address the effects of COVID-19 on food security [18].

We thank all of the contributors to these two Special Issues and hope that these publications will stimulate other researchers to conduct research on the impact of the COVID-19 pandemic in LMICs and to publish their results. We hope that this will prepare us better for future pandemics.
